# Anaortic Off-Pump vs Conventional CABG: A Propensity-Matched Single-Center Experience

**DOI:** 10.1016/j.atssr.2026.02.026

**Published:** 2026-03-10

**Authors:** Mark R. Lutz, Shaelyn M. Cavanaugh, Samuel J. Martin, Karikehalli Dilip, Ahmad Nazem, Anton Cherney, Zhandong Zhou, Charles J. Lutz

**Affiliations:** 1 College of Medicine, SUNY Upstate Medical University, Syracuse, New York, USA; 2 Department of Surgery, University of Rochester Medical Center, Rochester, New York, USA; 3 Department of Cardiac Surgery, St. Joseph’s Hospital Medical Center, Syracuse, New York, USA

## Abstract

**Background:**

Obesity is associated with increased perioperative risk in coronary artery bypass grafting (CABG), including higher rates of stroke, bleeding, and prolonged recovery. Anaortic, off-pump CABG, avoiding cardiopulmonary bypass and aortic manipulation, may mitigate these risks. We evaluated outcomes of anaortic off-pump CABG vs CABG with aortic manipulation in obese patients with 3-vessel disease.

**Methods:**

We conducted a retrospective, single-center study of obese patients (body mass index >30 kg/m^2^) with 3-vessel coronary artery disease who were undergoing isolated CABG. Patients were stratified by surgical technique: anaortic off-pump CABG vs CABG with aortic manipulation. Propensity score matching (1:2) was performed to adjust for baseline differences. The primary outcome was 30-day death or stroke. Secondary outcomes included transfusion, operative time, and major postoperative complications.

**Results:**

After propensity matching, 926 patients were included (320 with anaortic CABG; 606 with CABG with aortic manipulation). The anaortic group had significantly lower rates of 30-day death or stroke (0.9% vs 3.6%; *P* = .028), shorter operative times (293 minutes vs 352 minutes; *P* < .001), and fewer intraoperative transfusions (8.2% vs 25.0%; *P* < .001). When assessed relative to The Society of Thoracic Surgeons predicted risk, the anaortic strategy was associated with lower observed-to-expected mortality (0.31 vs 0.99; *P* = .032) and stroke (0.00 vs 0.74; *P* = .018). Other postoperative complications were similar between groups.

**Conclusions:**

In obese patients with multivessel coronary artery disease, anaortic off-pump CABG was associated with reduced rates of early death or stroke, fewer transfusions, and shorter operative times. These findings support the use of the anaortic technique and highlight its potential role in optimizing outcomes in the growing population of obese CABG candidates.

## Visual Abstract

**Figure:**
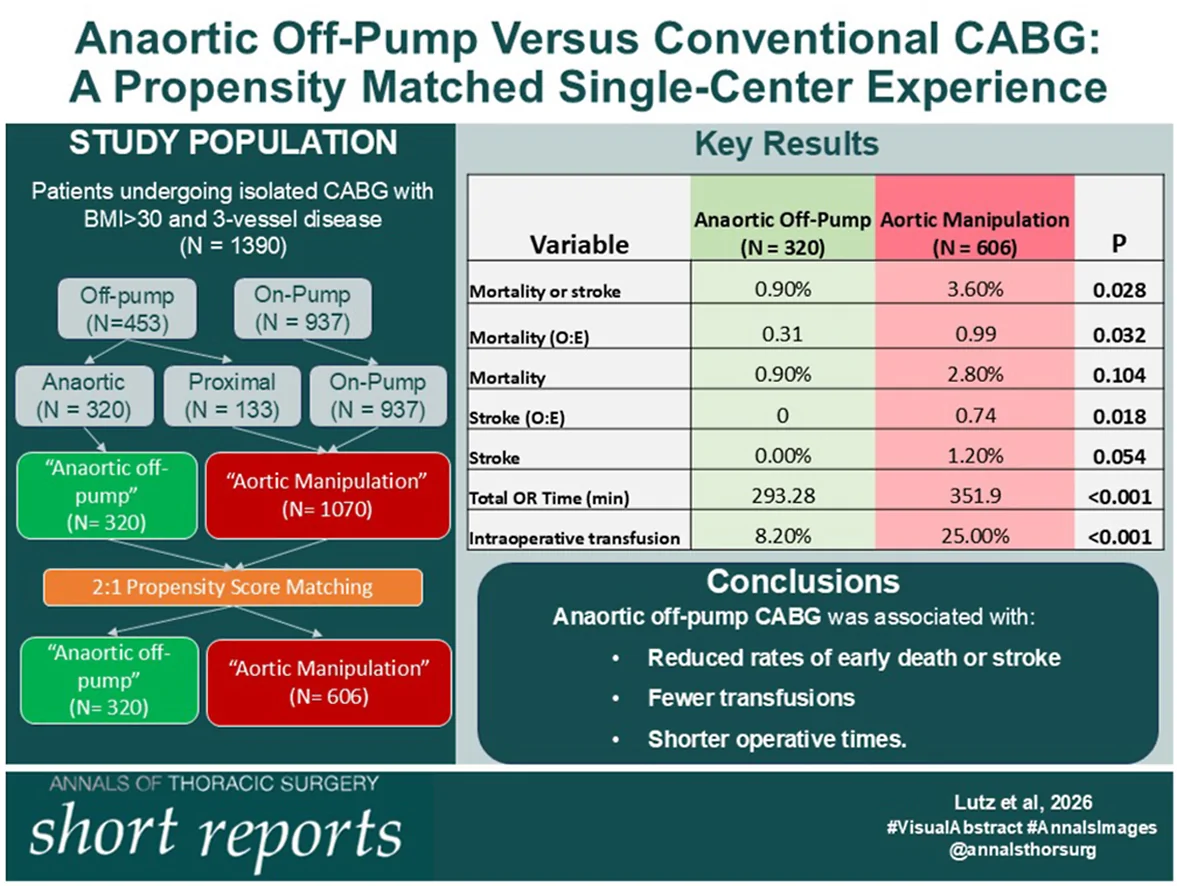

**In Short**

▪ Anaortic, off-pump CABG in obese patients with 3-vessel disease was associated with significantly lower 30-day death or stroke compared with CABG involving aortic manipulation after propensity matching.
▪ O/E mortality and stroke were both significantly reduced with the anaortic strategy.
▪ Anaortic CABG also resulted in shorter operative times and fewer transfusions without increased postoperative complications.

Multiarterial grafting (MAG) has become a major focus in the evolution of coronary artery bypass grafting (CABG), with recent long-term data showing improved survival compared with single-arterial grafting.^1^ Reflecting this survival advantage, The Society of Thoracic Surgeons (STS) Adult Cardiac Surgery Database reported a 30% increase in MAG between 2018 and 2023, reaching 17.9% of isolated CABG cases in 2023.^2^ Simultaneously, the field has witnessed rising the adoption of minimally invasive cardiac surgery CABG approaches, along with a modest resurgence of off-pump CABG. In 2023, off-pump surgery was performed in 11.5% of isolated CABG cases (18,656 of 161,907), a finding reflecting continued interest in techniques that reduce physiologic burden and recovery time.^2^

Obesity is a common comorbidity in patients undergoing CABG and is associated with increased perioperative risks, including stroke, bleeding, and prolonged recovery.^3^ Traditional cardiopulmonary bypass may exacerbate these risks through systemic inflammation and embolic events. In this context, anaortic, off-pump CABG, which avoids both cardiopulmonary bypass and aortic manipulation, has gained interest as a strategy to mitigate perioperative complications, particularly stroke and transfusion-related morbidity.

In this study, we examined early outcomes of anaortic, off-pump CABG vs CABG involving aortic manipulation in obese patients with 3-vessel coronary artery disease (CAD), with a focus on perioperative stroke, mortality, transfusion, and operative efficiency.

## Patients and Methods

Using institutional STS Adult Cardiac Surgery Database data, we conducted a retrospective, cohort study of adult patients who underwent isolated CABG at St. Joseph’s Health Hospital in Syracuse, New York, between January 2014 and March 2024. Patients were included if they had a body mass index (BMI) >30 kg/m^2^ and angiographically confirmed 3-vessel CAD. Those patients undergoing concomitant valve or aortic procedures were excluded.

The anaortic group consisted of patients who underwent CABG without the use of cardiopulmonary bypass and without any manipulation of the ascending aorta. These procedures relied exclusively on in situ arterial or composite graft configurations, with no proximal anastomoses to the aorta or use of proximal anastomotic sealing devices. The aortic manipulation group included patients who underwent either on-pump CABG or off-pump CABG requiring full or partial clamping of the aorta, for arterial cannulation or proximal anastomoses, respectively. The choice of operative approach and conduit configuration was made at the discretion of the attending surgeon.

Propensity scores were estimated using a logistic regression model incorporating the following preoperative variables: sex, age, BMI, history of cerebrovascular disease, and STS predicted risk of mortality. Patients undergoing anaortic, off-pump CABG were matched 1:2 to patients undergoing CABG with aortic manipulation by using nearest-neighbor matching without replacement and a caliper width of 0.2. All 320 patients in the anaortic, off-pump CABG cohort were successfully matched to 606 patients in the aortic manipulation cohort; 463 unmatched control patients were excluded from the final analysis. Covariate balance was assessed using standardized mean differences (SMDs) and visual inspection of propensity score distributions. After matching, all covariates demonstrated absolute SMDs <0.1, indicating acceptable balance between groups (Figure).

**Figure.**
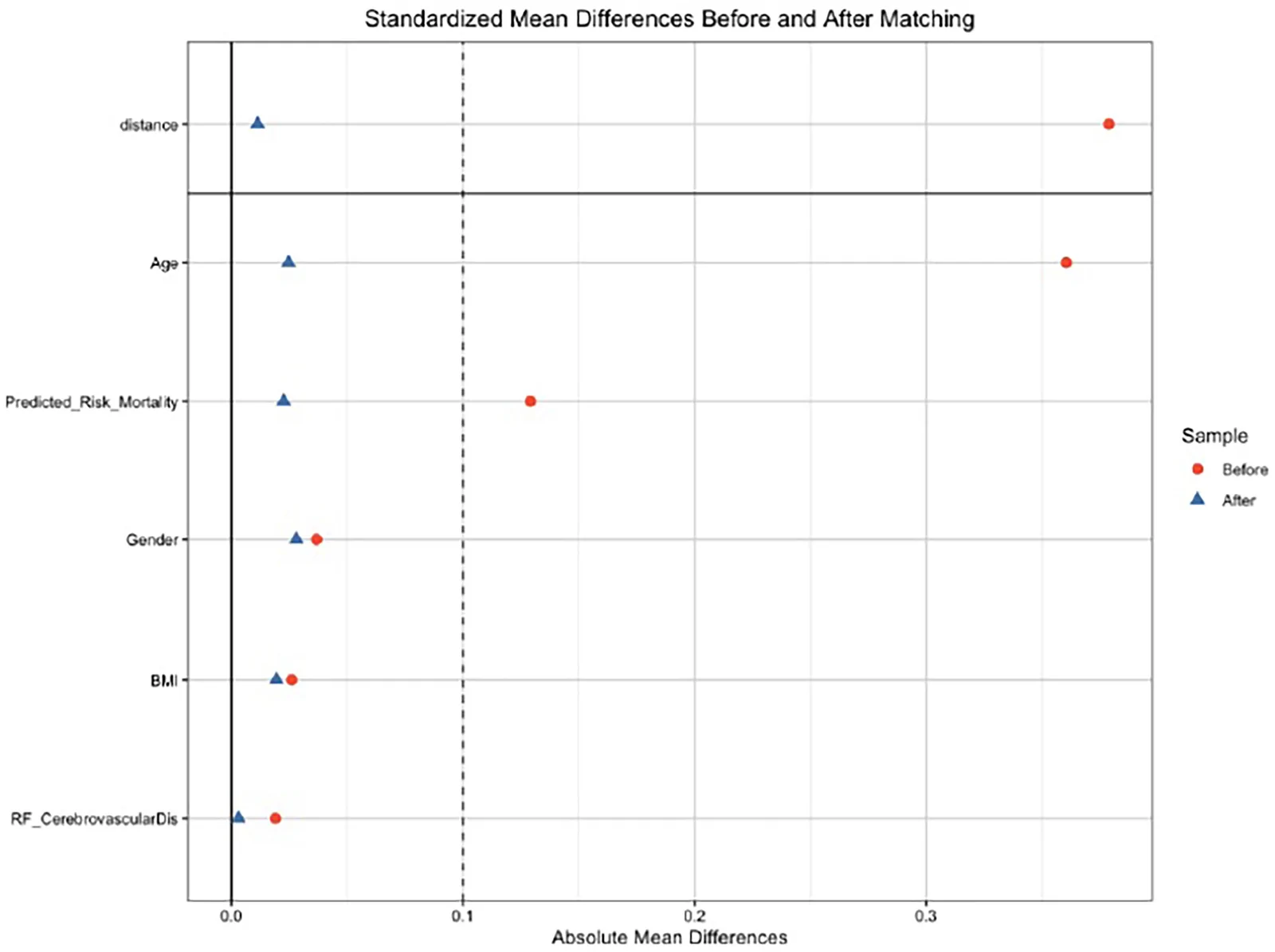
Standard mean distance before and after propensity matching. (BMI, body mass index; Dis, disease; RF, risk factor.)

The primary outcome was the composite of 30-day mortality or stroke. Secondary outcomes included operative time, transfusion requirements, length of stay, and major postoperative complications. Observed-to-expected (O/E) ratios for mortality and stroke were calculated using STS-predicted risks, and group differences were assessed using χ^2^ testing. Continuous variables were compared using 2-sample *t* tests, and categorical variables were compared using χ^2^e or Fisher exact tests, as appropriate. A 2-sided *P* value <.05 was considered statistically significant. Statistical analyses were performed using SPSS software version 29.0.2 (IBM Corp).

## Results

After propensity matching, 926 patients with BMI >30 kg/m^2^ and 3-vessel CAD were included in the analysis, comprising 320 patients who underwent anaortic, off-pump CABG and 606 patients who underwent CABG with aortic manipulation.

Baseline demographic and clinical characteristics of the matched cohort are summarized in Table 1. There were no clinically meaningful differences between groups with respect to age, sex, BMI, diabetes, renal dysfunction, cerebrovascular disease, STS-predicted risk of mortality, or predicted stroke risk.

**Table 1:** Comparison of Patients’ Characteristics Between Coronary Artery Bypass Grafting Techniques

Preoperative Characteristics	CABG Technique		*P*
	Anaortic Off-Pump	Aortic Manipulation	
	(n = 320)	(n = 606)	
Age, y	69.00 (61.00-77.00)	68.00 (61.00-74.0)	.155
BMI, kg/m^2^	33.78 (31.61-37.39)	34.00 (32.00-37.42)	.845
Race: White	266 (97.1)	576 (95.0)	.109
Sex: male	228 (71.2)	424 (70.0)	.741
Diabetes	208 (65.0)	370 (61.1)	.268
Renal failure	9 (2.8)	14 (2.3)	.806
Hypertension	292 (91.2)	558 (92.1)	.756
Tobacco use	…	…	.299
Former	154 (48.1)	301 (49.7)	
Current	43 (13.4)	99 (16.3)	
Illicit drug use	4 (1.3)	17 (2.8)	.198
Liver disease	8 (2.5)	12 (2.0)	.673
Immunocompromise	12 (3.8)	12 (2.0)	.163
Peripheral artery disease	50 (15.6)	81 (13.4)	.402
Cerebrovascular disease	98 (30.6)	185 (30.5)	1.000
Previous cardiac intervention	152 (47.5)	248 (40.9)	.064
Heart failure	126 (39.4)	230 (38.0)	.725
Cardiac arrhythmia	79 (24.7)	106 (17.5)	**.012**
Incidence			.607
1st operation	306 (95.6)	586 (96.7)	
1st reoperation	14 (4.4)	19 (3.1)	
2nd reoperation	0 (0.0)	0 (0.0)	
3rd reoperation	0 (0.0)	1 (0.2)	
STS stroke	1.55 [1.39]	1.56 [1.32]	.974
STS PROM	3.01 [4.67]	2.84 [4.99]	.609
Preoperative EF	51.15 [11.32]	51.84 [11.57]	.390

Data reported as mean [SD], median (interquartile range), or count (%).

*P* < .05 in bold.

BMI, body mass index; CABG, coronary artery bypass grafting; EF, ejection fraction; PROM, predicted risk of mortality; STS, The Society of Thoracic Surgeons.

Operative characteristics and postoperative outcomes are presented in Table 2. MAG was used significantly more often in the anaortic group (51.2% vs 34.5%; *P* < .001). Total mean operative time was shorter in the anaortic cohort (293.3 [52.3] minutes vs 351.9 [72.0] minutes; *P* < .001), and intraoperative transfusion was required less frequently (8.2% vs 25.0%; *P* < .001).

**Table 2:** Comparison of Operative and Postoperative Variables Between Coronary Artery Bypass Grafting Techniques

Operative Variables and Postoperative Outcomes	CABG Technique		*P*
	Anaortic Off-Pump (n = 320)	Aortic Manipulation (n = 606)	
Operative variables			
Status			**<.001**
Elective	145 (45.3)	209 (34.5)	
Urgent	168 (52.5)	372 (61.4)	
Emergency	7 (2.2)	25 (4.1)	
Multiarterial CABG	164 (51.2)	209 (34.5)	**<.001**
Cardiopulmonary bypass time, min	N/A	96.97 [44.61]	N/A
Aortic cross-clamp time, min	N/A	74.60 [20.80]	N/A
Total OR time, min	293.28 [52.27]	351.90 [71.98]	**<.001**
Intraoperative transfusion	26 (8.2)	151 (25.0)	**<.001**
Postoperative outcomes			
**Death or stroke (30 d)**	3 (0.9)	22 (3.6)	**.028**
**Mortality (O/E)**	3/9.66 (0.31)	17/17.21 (0.99)	**.032**
**Stroke (O/E)**	0/4.99 (0.00)	7/9.45 (0.74)	**.018**
Mortality (30 d)	3 (0.9)	17 (2.8)	.104
Stroke (30 d)	0 (0.0)	7 (1.2)	.054
Postoperative LOS, d	7.11 [6.63]	8.81 ([.32])	**.004**
Readmission	25 (8.2)	53 (9.1)	.742
Surgical site infection	3 (0.9)	7 (1.2)	.761
Reoperation for bleeding	3 (0.9)	11 (1.8)	.298
Pneumonia	7 (2.2)	25 (4.1)	.125
Transfusion required	37 (11.6)	147 (24.3)	**<.001**
Renal failure	5 (3.7)	24 (4.0)	.637
Postoperative EF, %	46.83 [11.89]	46.60 [13.12]	.941

Data reported as count (%), mean [SD], or n/n (O/E).

*P* < .05 in bold.

CABG, coronary artery bypass grafting; EF, ejection fraction; LOS, length of stay; O/E, observed to expected ratio; N/A, not applicable; OR, operating room.

The primary composite end point of 30-day death or stroke occurred significantly less often in the anaortic group (0.9% vs 3.6%; *P* = .028). When assessed individually, 30-day mortality was numerically lower in the anaortic cohort (0.9% vs 2.8%; *P* = .104), and stroke occurred in 0% of anaortic patients compared with 1.2% in the aortic manipulation group (*P* = .054).

When outcomes were evaluated relative to STS-predicted risk, the anaortic group demonstrated a significant reduction in both mortality and stroke. Observed mortality in the anaortic cohort was 3 deaths compared with 9.66 expected (O/E = 0.31), whereas the aortic manipulation group experienced 17 observed deaths compared with 17.21 expected (O/E = 0.99; *P* = .032). Similarly, no strokes were observed in the anaortic group despite 4.99 expected events (O/E = 0.00), compared with 7 observed strokes and 9.45 expected in the aortic manipulation group (O/E = 0.74; *P* = .018) (Table 2).

In the anaortic group, mean postoperative length of stay was shorter (7.11 [6.63] days vs 8.81 [9.32] days; *P* = .004), and postoperative transfusion requirements were significantly lower (11.6% vs 24.3%; *P* < .001). Rates of readmission, surgical site infection, reoperation for bleeding, pneumonia, renal failure, and postoperative ejection fraction were similar between groups.

## Comment

In this retrospective cohort of obese patients with 3-vessel CAD who underwent CABG, the use of an anaortic, off-pump strategy was associated with a significantly lower incidence of the composite end point of 30-day death or stroke. Notably, when outcomes were assessed against expected event rates using STS risk models, the anaortic group demonstrated a substantial reduction in both mortality (O/E 0.31 vs 0.99; *P* = .032) and stroke (O/E 0.00 vs 0.74; *P* = .018).These findings suggest that avoidance of aortic manipulation may confer important early protective effects in obese CABG patients.

These findings align with a growing body of literature that positions anaortic CABG as a powerful strategy for neurologic protection. A 2017 network meta-analysis by Zhao and colleagues^4^ demonstrated a stepwise reduction in stroke risk with decreasing levels of aortic manipulation, with anaortic off-pump CABG reducing stroke by up to 78% compared with traditional on-pump CABG. Although the magnitude of this effect varies across studies, the cumulative evidence supports the central premise: minimizing aortic manipulation reduces embolic risk and improves perioperative safety.

Importantly, the early stroke risk observed in CABG has long been recognized as a disadvantage relative to percutaneous coronary intervention. A 2018 pooled analysis by Head and colleagues^5^ found that stroke rates were significantly higher in the first 30 days after CABG, particularly among patients with multivessel disease. Critically, 5-year mortality was markedly higher among patients who experienced a stroke in that early period.^5^ Our finding of no observed strokes in the anaortic group despite a predicted stroke burden reinforces the importance of surgical technique in minimizing neurologic injury and challenges the notion that CABG-associated stroke is an unavoidable consequence of the operation.

These findings align with current clinical guidelines that emphasize the importance of minimizing aortic manipulation during CABG. The European Society of Cardiology and the European Association for Cardio-Thoracic Surgery recommend minimizing aortic manipulation as a class IB indication and explicitly support the use of anaortic, off-pump techniques in patients with a calcified or diseased ascending aorta.^6^ A 2020 American Heart Association scientific statement on perioperative stroke prevention also emphasized anaortic off-pump CABG as a foundational technique to reduce neurologic complications in cardiac surgery.^7^ Our results support these recommendations and suggest that the benefits of anaortic techniques can extend to a broader population of patients requiring coronary revascularization.

Although anaortic, off-pump CABG has traditionally been reserved for high-risk patients with calcified aortas or significant comorbidities, there is growing momentum to broaden its use. Vallely and colleagues^8^ argued that the time has come to move beyond viewing anaortic, off-pump CABG as a niche technique and instead recognize it as a foundational strategy for improving neurologic outcomes across the spectrum of surgical patients. The principal barrier to widespread adoption remains perceived technical difficulty; however, mastery of technically demanding procedures is common across cardiac surgery, especially in an era of increasing minimally invasive cardiac surgery techniques. Anaortic CABG, with its potential to reduce stroke, bleeding, and even mortality, offers a clear return on that investment.

Our findings further suggest that anaortic CABG is not only feasible but also highly compatible with other advanced strategies, particularly MAG. In our cohort, MAG was used significantly more often in the anaortic group, a finding reflecting both institutional practice and national trends. According to the STS Adult Cardiac Surgery Database, MAG use has increased by >30% between 2018 and 2023, thus reflecting recent data showing improved long-term survival benefits and underscoring a shift toward durability-focused revascularization.^1^^,^^2^ The anaortic approach complements this trend by enabling composite graft construction without the need for proximal aortic anastomoses. This technical synergy becomes even more critical as the field moves toward minimally invasive and robotic-assisted CABG, where access to the ascending aorta is even more limited. In that context, the ability to perform total-arterial, anaortic revascularization is not just an advanced skill—it is a prerequisite for delivering high-quality surgical revascularization through less invasive means. Our findings highlight that anaortic and MAG techniques are not mutually exclusive but are, in fact, integral components of a modern, adaptable coronary surgery strategy.

In conclusion, in obese patients with multivessel CAD, anaortic off-pump CABG was associated with reduced rates of early death or stroke despite higher preoperative risk, fewer transfusions, and shorter operative times compared with CABG with aortic manipulation. These findings support the broader adoption of this approach as a feasible strategy that provides excellent early outcomes, facilitates MAG and aligns with the future direction of minimally invasive coronary surgery.
